# Investigation of Sevoflurane-Induced Apoptotic Damage in Human Cardiomyocytes and the Protective Efficacy of Ascorbic Acid

**DOI:** 10.3390/medicina62050945

**Published:** 2026-05-12

**Authors:** Eyüp Aydoğan, İshak Suat Övey, Oğuz Karahan

**Affiliations:** 1 Department of Anesthesiology and Reanimation, Faculty of Medicine, Alanya Alaaddin Keykubat University, 07425 Alanya, Türkiye; eyup.aydogan@alanya.edu.tr; 2 Department of Physiology, Faculty of Medicine, Alanya Alaaddin Keykubat University, 07425 Alanya, Türkiye; 3 Department of Cardiovascular Surgery, Faculty of Medicine, Alanya Alaaddin Keykubat University, 07425 Alanya, Türkiye; oguz.karahan@alanya.edu.tr

**Keywords:** sevoflurane, TRPV1, cardiomyocyte, ascorbic acid, apoptosis

## Abstract

*Background and Objectives:* Sevoflurane, a widely used volatile anesthetic, can induce oxidative stress and apoptosis, but the underlying mechanisms in human cardiomyocytes remain unclear. This study investigated the role of transient receptor potential vanilloid 1 (TRPV1) channels in sevoflurane-induced cardiotoxicity and the potential mitigating effect of ascorbic acid. *Materials and Methods:* Human cardiomyocytes were exposed to sevoflurane (5.1%, 6 h) and/or ascorbic acid (1 mM, 30 min), with or without the TRPV1 channel antagonist capsazepine and with the TRPV1 channel agonist Capsaicin. Intracellular calcium, reactive oxygen species (ROS), apoptosis, mitochondrial membrane potential, and caspase-3/9 activities were assessed. *Results:* Sevoflurane significantly increased intracellular calcium levels, ROS production, mitochondrial depolarization, apoptosis, and caspase-3/9 activity compared with controls (*p* < 0.001). These effects were attenuated by capsazepine, suggesting a role for TRPV1 involvement. Ascorbic acid pretreatment significantly reduced sevoflurane-induced elevations in all parameters (*p* < 0.001). Combined ascorbic acid and capsazepine treatment yielded further reductions in calcium, ROS, apoptosis, and caspase activities compared to ascorbic acid alone (*p* < 0.05). *Conclusions:* Sevoflurane induces apoptosis in human cardiomyocytes via ROS-mediated activation of the TRPV1 channel, leading to calcium overload, mitochondrial dysfunction, and caspase-dependent cell death. Ascorbic acid exerts mitigating effects by reducing oxidative stress and modulating TRPV1 channel activity, suggesting a potential therapeutic strategy for myocardial protection during sevoflurane anesthesia.

## 1. Introduction

Sevoflurane is a volatile anesthetic agent widely used in clinical anesthesia practice. Although it is preferred, particularly in cardiac surgery, due to its pharmacokinetic superiority and cardioprotective preconditioning effects, recent studies have shown that the biological effects of sevoflurane are not always protective; under certain conditions, it can cause cytotoxicity and apoptosis through oxidative stress [1]. Piao et al. revealed that Sevoflurane exposure triggers cell death through DNA damage and apoptosis by increasing intracellular reactive oxygen species (ROS) levels in neuronal cells. These findings suggest that similar risks may exist for the myocardium, a metabolically highly active tissue susceptible to oxidative damage [2]. The exact molecular mechanisms by which sevoflurane-induced oxidative stress initiates apoptosis in cardiomyocytes remain unclear. In recent years, it has become known that transient receptor potential (TRP) ion channels are sensors that respond particularly to stimuli such as oxidative stress and inflammation. TRPV1 (Transient Receptor Potential Vanilloid 1), a calcium channel from this family, can be directly activated by reactive oxygen species and carbonyl species [3]. Activation of TRPV1, which is expressed in cardiomyocytes, can disrupt intracellular calcium homeostasis, leading to mitochondrial dysfunction and apoptosis. In this context, the hypothesis that sevoflurane-enhanced ROS can trigger apoptosis in cardiomyocytes by stimulating TRPV1 channels is gaining strength. Strengthening cellular defense mechanisms against oxidative stress is a promising strategy in preventing anaesthetic-induced myocardial damage. Vitamin C (ascorbic acid), a potent antioxidant, is known for its direct scavenging of free radicals and its contribution to the regeneration of other antioxidants. Ludke et al. demonstrated a protective effect of vitamin C against doxorubicin-induced cardiotoxicity at the subcellular level, revealing its role in maintaining mitochondrial integrity and reducing oxidative damage [4]. Similarly, vitamin C treatment in human pluripotent stem cell-derived cardiomyocytes reduced intracellular ROS levels, maintained cellular homeostasis, and exhibited anti-aging effects. These findings clearly support the protective potential of vitamin C against oxidative stress in cardiomyocytes [5].

We aimed to comprehensively investigate the role of TRPV1 channels in mediating the apoptotic effects of sevoflurane in cardiomyocyte cells, as well as to elucidate the underlying molecular mechanisms by which ascorbic acid attenuates reactive oxygen species (ROS) levels, thereby evaluating the potential protective and modulatory effects of TRPV1 channel activity on oxidative stress-induced cellular injury.

## 2. Materials and Methods

### 2.1. Reagents/Stains

Human Cardiomyocyte Complete Media with Serum and Human Cardiomyocyte Serum Free Media were procured from Celprogen (Torrance, CA, USA), Trypsin–EDTA, dimethyl sulfoxide, and Dihydrorhodamine-123 (DHR 123) were procured from Sigma Aldrich (St. Louis, MO, USA). Caspase 3 (AC-DEVD-AMC) and 9 (AC-LEHD-AMC) substrates were procured from Enzo (Lausanne, Switzerland). APO percentage assay with a release buffer was procured from Biocolor (Belfast, Northern Ireland, UK). Fura 2 (AM) fluorescent calcium stain was procured from Calbiochem (Darmstadt, Germany). Pluronic^®^ F-127 was procured from Biovision (San Francisco, CA, USA). Probenecid, and a mitochondrial stain 5.50, 6.60-tetrachloro-1.10.3.30-tetraethylbenzimidazolylcarbocyanine iodide (JC-1) was procured from Santa Cruz (Dallas, TX, USA).

### 2.2. Cell Culture

**Figure 1:**
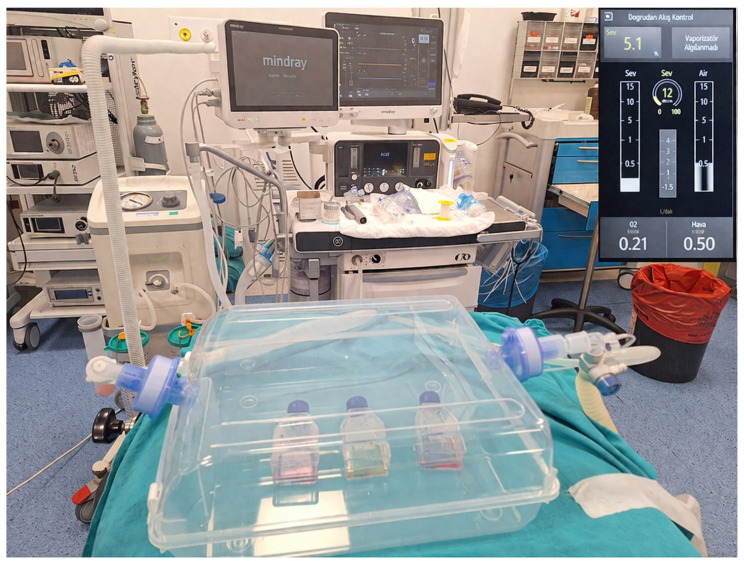
Anesthesia device and airtight chamber system used during 5.1% sevoflurane exposure of cardiomyocyte Cells.

Human cardiomyocyte cells were obtained from Celprogen Inc. (Cat. Number: 36044-15; Celprogen, Torrance, CA, USA). Cells were cultured in Human Cardiomyocyte Complete Media with Serum (Commercial form includes serum and antibiotics). Cultures were maintained in T25 flasks (Human Cardiomyocyte Primary Cell Extracellular Matrix, Cat. Number E36044-15-T25, Celprojen, Torrance, CA, USA) and incubated at 37 °C in a humidified atmosphere with 5% CO_2_. At 75–85% confluence, cells were treated with the compounds described in the experimental groups section. All experimental groups were maintained under identical physical conditions, including temperature, CO_2_, and O_2_ levels, during the exposure period. In addition, a matched control group was kept in the same sealed-chamber system without sevoflurane exposure. The cells were exposed to 5.1% sevoflurane, % 21 oxygen, 0.04% CO_2_ for 6 h in a sealed, transparent container with a dual respiratory circuit connected at the inlet and outlet. A gas sampling line was connected to the outlet circuit to continuously monitor the constant volatile anesthetic concentration (Figure 1). The incubation case has been heated to the target temperature via an electronically regulated heating device from the base. Temperature changes have been closely monitored and corrected using a laser thermometer. A Mindray A9 anesthesia machine from Shenzhen, China, 2025, was used in the study. Following treatment, cells were washed, detached using 0.25% trypsin–EDTA, collected into 15 mL tubes, and centrifuged at 100× *g* for 5 min. After washing with fresh medium, cells were prepared for subsequent experiments.

#### 2.2.1. Study Groups

HCCs (Human Cardiomyocyte Cells) were cultured at 37 °C. The cells were divided into seven main groups.

**Group 1** (Control)**:** After 75–85% confluence, similar to the other groups, HCCs were maintained under similar physical conditions in the operating room without exposure to sevoflurane or ascorbic acid.

**Group 2** (Sevo)**:** HCCs in the group were incubated with 5.1% sevoflurane for 6 h [6].

**Group 3** (Sevo + Cpz)**:** HCCs in the group were incubated with 5.1% sevoflurane for 6 h. and then incubated with capsazepine (TRPV1 channels antagonist, CPZ, 0.1 mM, 30 min).

**Group 4** (AA)**:** HCCs in the group were incubated with 1 mM ascorbic acid for 30 min [7].

**Group 5** (AA + Cpz)**:** HCCs in the group were incubated with 1 mM ascorbic acid for 30 min. and then incubated with capsazepine (TRPV1 channels antagonist, CPZ, 0.1 mM, 30 min).

**Group 6** (Sevo + AA)**:** HCCs in the group were incubated with 1 mM ascorbic acid for 30 min. and then incubated with 5.1% sevoflurane for 6 h.

**Group 7** (Sevo + AA + Cpz)**:** HCCs in the group were incubated with 1 mM ascorbic acid for 30 min. and then incubated with 5.1% sevoflurane for 6 h. and then incubated with 0.1 mM capsazepine for 30 min.

During calcium signaling analysis (Fura-2/AM), cardiomyocyte cells were stimulated on the 20th cycle with 0.1 mM Cap in the presence of 1.2 mM calcium and calcium-free buffer in an extracellular environment. For apoptosis, intracellular reactive oxygen species, mitochondrial depolarization, caspase-3 and caspase-9 experiments, the HCCs were further treated with TRPV1 channel agonist Capsaicin (Cap, 0.1 mM, 10 min) for activation of the TRPV1 channel before related analysis.

#### 2.2.2. Measurement of Intracellular Free-Calcium Concentration ([Ca^2+^]i)

Intracellular Ca^2+^ levels were measured using the UV-excitable calcium indicator Fura-2 acetoxymethyl ester (Fura-2-AM). Fluorescence emission at 510 nm was recorded at alternating excitation wavelengths of 340/380 nm every 3 s using a microplate reader (Synergy™ H1, Biotek, Winooski, VT, USA) following stimulation with Capsaicin (Cap, 0.1 mM). Measurements were performed as previously described. The experiments were conducted by combining the methods described in the studies of Uğuz and Martínez [8,9].

#### 2.2.3. Apoptosis and Intracellular ROS Production

Apoptosis was assessed using the APOPercentage™ assay kit (Biocolor Ltd., Belfast, Northern Ireland, UK) according to the manufacturer’s instructions. This dye-uptake assay selectively stains apoptotic cells due to phosphatidylserine externalization. Following treatment, apoptotic human cardiomyocyte cells were quantified spectrophotometrically at 550 nm using a microplate reader (Synergy™ H1, Biotek, Winooski, VT, USA) as described previously [10,11]. Intracellular ROS production was evaluated using Rhodamine 123 (Rh123). Fluorescence intensities were measured at excitation/emission wavelengths of 488/543 nm using the same microplate reader [10,11].

#### 2.2.4. Caspase-3 and Caspase-9 Activity Assays

Caspase-3 and caspase-9 activities were determined using fluorogenic substrates Ac-DEVD-AMC and Ac-LEHD-AMC, respectively, as previously reported [10,11]. Fluorescence was measured at 360 nm excitation and 460 nm emission using a microplate reader (Synergy™ H1, Biotek, Winooski, VT, USA).

#### 2.2.5. Mitochondrial Membrane Potential (JC-1) Analysis

Mitochondrial membrane potential (ΔΨm) was assessed using JC-1 dye (1 µM). Cardiomyocyte cells were incubated with JC-1 at 37 °C for 45 min in the dark. After incubation, cells were washed with PBS to remove excess dye. Fluorescence intensity was measured using a fluorescence microplate reader (Synergy™ H1, BioTek, Winooski, VT, USA). JC-1 monomer fluorescence was recorded at 485/535 nm (green), and JC-1 aggregate fluorescence was recorded at 535/590 nm (red). Mitochondrial membrane potential was quantified as the ratio of red to green fluorescence intensity (590/535) for each sample. Background fluorescence was subtracted prior to analysis. The obtained red/green ratios were normalized to the mean value of the control group and expressed as relative changes (% of control). A decrease in the red/green fluorescence ratio was interpreted as mitochondrial depolarization [10,11].

#### 2.2.6. Statistical Analyses

All results were expressed as means ± standard deviation (SD). Normality of data distribution was assessed, and homogeneity of variances was evaluated using both Bartlett’s and Brown–Forsythe tests. Statistical significance among the groups was determined using one-way analysis of variance (ANOVA), followed by Tukey’s post hoc test for multiple comparisons. Statistical analyses were performed using GraphPad Prism version 7.04 for Windows (GraphPad Software, San Diego, CA, USA). A *p*-value < 0.05 was considered statistically significant, corresponding to a 95% confidence interval.

## 3. Results

### 3.1. Effects of Sevoflurane and Ascorbic Acid on Cytosolic Calcium Levels in Human Cardiomyocyte Cells (HCCs)

**Figure 2:**
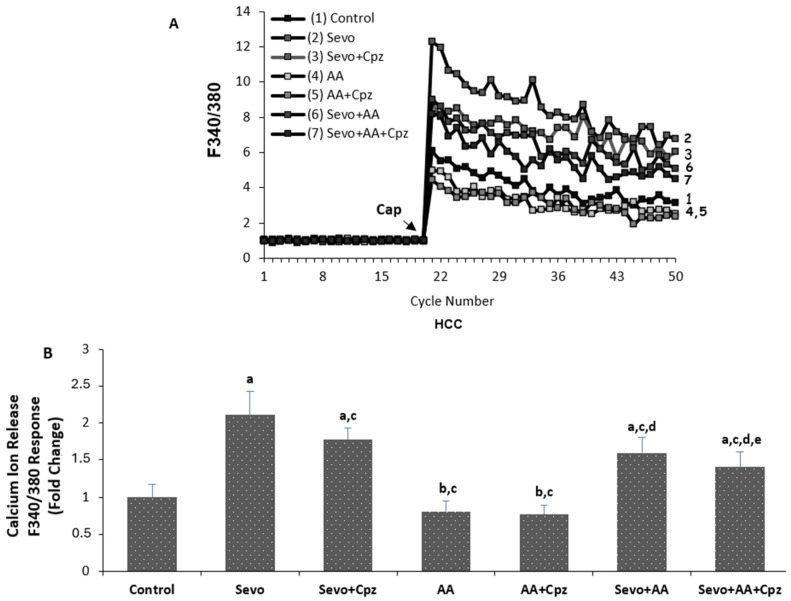
(**A**,**B**) The effect of sevoflurane (Sevo, 5.1%, 6 h) and ascorbic acid (AA, 1 mM, 30 min.) on cytosolic calcium levels in cardiomyocyte cells. Cells are stimulated by Capsaicin (Cap 0.1 mM and on 20th cycle) but they are inhibited by capsazepine (Cpz 0.1 mM for 30 min) (mean ± SD and *n* = 3). ^a^ *p* ˂ 0.001 and ^b^ *p* ˂ 0.05 vs. control, ^c^ *p* ˂ 0.001 vs. Sevo, ^d^ *p* ˂ 0.001 vs. AA and ^e^ *p* ˂ 0.05 vs. Sevo + AA group.

The effects of sevoflurane and ascorbic acid administrations on cytosolic calcium levels in HCCs are shown in Figure 2A,B. The TRP Vanilloid 1 channel stimulator Capsaicin and blocker capsazepine were used to evaluate intracellular Ca^2+^ increase through TRPV1 channels in the human cardiomyocyte cells. As shown in Figure 2B, the Ca^2+^ concentration in HCCs was greater in the sevoflurane and sevoflurane + ascorbic acid groups (*p* < 0.001) and was lower in the ascorbic acid group in comparison to the control group (*p* < 0.05). The Ca^2+^ level was lower in the sevoflurane + ascorbic acid and ascorbic acid groups in comparison to the sevoflurane group (*p* < 0.001), but at the same time, the ascorbic acid group was lower than the sevoflurane + ascorbic acid (*p* < 0.001). When sevoflurane was compared with sevoflurane + capsazepine and sevoflurane + ascorbic acid was compared with the sevoflurane + ascorbic acid + capsazepine groups, the groups using capsazepine channel inhibitor had lower cytosolic calcium levels than the groups not using capsazepine (*p* < 0.001 and *p* < 0.05) and there was no statistically significant difference between the ascorbic acid and ascorbic acid + capsazepine groups.

### 3.2. Effects of Sevoflurane and Ascorbic Acid on Programmed Cell Death, Intracellular Reactive Oxygen Species (ROS), and Mitochondrial Depolarization Levels in Human Cardiomyocyte Cells (HCCs)

**Figure 3:**
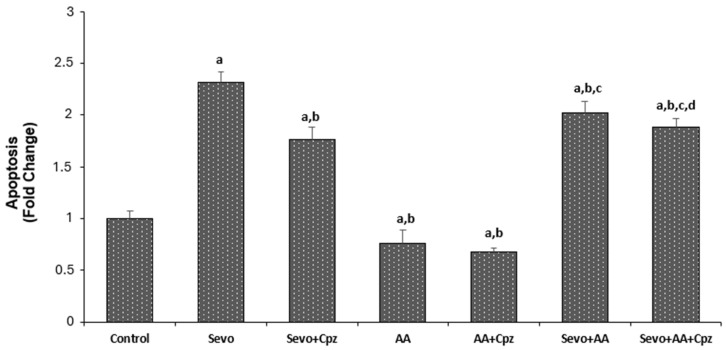
The effect of sevoflurane (Sevo, 5.1%, 6 h) and ascorbic acid (AA, 1 mM, 30 min.) on apoptosis levels in the cardiomyocyte cells. Cells are stimulated by Capsaicin (Cap 0.1 mM for 10 min) but they are inhibited by capsazepine (Cpz 0.1 mM for 30 min) (mean ± SD and *n* = 10). ^a^ *p* ˂ 0.001 vs. control, ^b^ *p* ˂ 0.001 vs. Sevo, ^c^ *p* ˂ 0.001 vs. AA and ^d^ *p* ˂ 0.05 vs. Sevo + AA.

**Figure 4:**
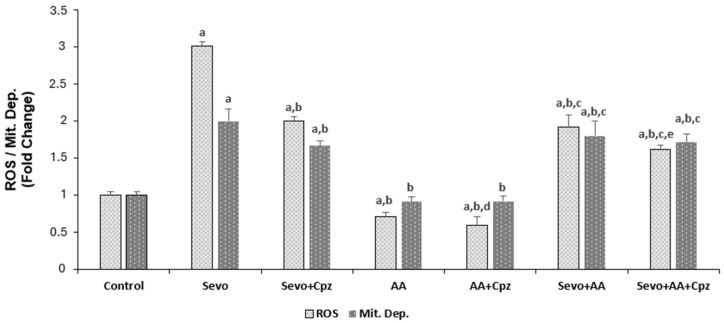
The effect of sevoflurane (Sevo, 5.1%, 6 h) and ascorbic acid (AA, 1 mM, 30 min.) on reactive oxygen and mitochondrial depolarization levels in the cardiomyocyte cells. Cells are stimulated by Capsaicin (Cap 0.1 mM for 10 min) but they are inhibited by capsazepine (Cpz 0.1 mM for 30 min) (mean ± SD and *n* = 10). ^a^ *p* ˂ 0.001 vs. control, ^b^ *p* ˂ 0.001 vs. Sevo, ^c^ *p* ˂ 0.001 and ^d^ *p* ˂ 0.05 vs. AA and ^e^ *p* ˂ 0.001 vs. Sevo + AA.

The effects of sevoflurane and ascorbic acid administrations on apoptosis, ROS and mitochondrial depolarization levels in HCCs are shown in Figure 3 and Figure 4. Apoptosis, ROS and mitochondrial depolarization levels were greater in the sevoflurane and sevoflurane + ascorbic acid groups (*p* < 0.001) and the values were lower in all groups except for the mitochondrial depolarization analysis in the ascorbic acid group in comparison to the control group (*p* < 0.001). There was no statistically significant difference between the control and ascorbic acid groups in mitochondrial depolarization analysis. The programmed cell death, ROS and mitochondrial depolarization levels were lower in the sevoflurane + ascorbic acid and ascorbic acid groups in comparison to the sevoflurane group (*p* < 0.001). But at the same time, the ascorbic acid group was lower than the sevoflurane + ascorbic acid group when the sevoflurane + ascorbic acid and ascorbic acid groups were compared (*p* < 0.001). When sevoflurane was compared with sevoflurane + capsazepine and sevoflurane + ascorbic acid was compared with sevoflurane + ascorbic acid + capsazepine groups, the groups using capsazepine channel inhibitor had lower apoptosis, mitochondrial depolarization and ROS levels than the groups not using capsazepine (*p* < 0.001 and *p* < 0.05). When ascorbic acid groups were compared with ascorbic acid +capsazepine groups, the groups using capsazepine channel inhibitor had lower ROS levels than the groups not using capsazepine (*p* < 0.05) but there was no statistically significant difference between the ascorbic acid and ascorbic acid + capsazepine groups in apoptosis and mitochondrial depolarization levels.

### 3.3. Effects of Sevoflurane and Ascorbic Acid on Caspase 3 and Caspase 9 Levels in Human Cardiomyocyte Cells (HCCs)

**Figure 5:**
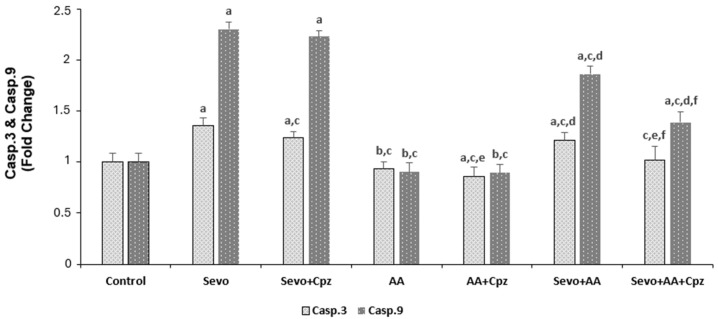
The effect of sevoflurane (Sevo, 5.1%, 6 h) and ascorbic acid (AA, 1 mM, 30 min.) on caspase 3 and caspase 9 levels in the cardiomyocyte cells. Cells are stimulated by Capsaicin (Cap 0.1 mM for 10 min) but they are inhibited by capsazepine (Cpz 0.1 mM for 30 min) (mean ± SD and *n* = 10). ^a^ *p* ˂ 0.001 and ^b^ *p* ˂ 0.05 vs. control, ^c^ *p* ˂ 0.001 vs. Sevo, ^d^ *p* ˂ 0.001 and ^e^ *p* ˂ 0.05 vs. AA and ^f^ *p* ˂ 0.001 vs. Sevo + AA.

The effects of sevoflurane and ascorbic acid administrations on caspase 3 and caspase 9 levels in HCCs are shown in Figure 5. In caspase 3 and caspase 9 analysis, sevoflurane and sevoflurane + ascorbic acid groups were greater (*p* < 0.001) and the ascorbic acid groups were lower in the comparison to the control group (*p* < 0.05). The caspase 3 and caspase 9 levels were lower in the sevoflurane + ascorbic acid and ascorbic acid groups in comparison to the sevoflurane group (*p* < 0.001). But at the same time, the ascorbic acid group was lower than the sevoflurane + ascorbic acid group when the sevoflurane + ascorbic acid and ascorbic acid groups were compared (*p* < 0.001). When sevoflurane was compared with sevoflurane + capsazepine and sevoflurane + ascorbic acid was compared with sevoflurane + ascorbic acid + capsazepine groups, the groups using capsazepine channel inhibitor had lower cytosolic calcium levels than the groups not using capsazepine (*p* < 0.001 and *p* < 0.05) in caspase 3 and caspase 9 analysis. When ascorbic acid was compared with ascorbic acid + capsazepine groups, the groups using capsazepine channel inhibitor had lower cytosolic calcium levels than the groups not using capsazepine (*p* < 0.001 and *p* < 0.05) in caspase 3 analysis (*p* < 0.05) but there was no statistically significant difference between the ascorbic acid and ascorbic acid + capsazepine groups in caspase 9 analysis.

## 4. Discussion

The present study provides the first evidence that sevoflurane induces apoptosis in human cardiomyocytes through oxidative stress-mediated activation of TRPV1 channels, and that ascorbic acid exerts its mitigating effects by modulating this pathway. Our findings demonstrate that sevoflurane exposure significantly increases intracellular calcium levels, reactive oxygen species (ROS) production, mitochondrial depolarization, and caspase-3/9 activities, all of which were markedly attenuated by TRPV1 antagonism with capsazepine. Furthermore, ascorbic acid pretreatment reduced these pathological parameters and partially reversed the protective effects when combined with capsazepine, suggesting that vitamin C acts, at least in part, through modulation of the TRPV1 channel. The finding that sevoflurane increases intracellular ROS and induces apoptotic cell death in human cardiomyocytes is consistent with previous reports in other cell types. Piao et al. demonstrated that sevoflurane exposure triggers parthanatos in neuronal cells through DNA damage mediated by increased ROS production [2]. Our study extends these observations to human cardiomyocytes, a cell type with high metabolic activity and susceptibility to oxidative injury. Importantly, we identified TRPV1 channels as critical mediators of this effect. The significant reduction in calcium influx, ROS levels, and apoptotic markers following capsazepine administration in sevoflurane-exposed cells confirms that TRPV1 activation is a key event in sevoflurane-induced cardiotoxicity (Figure 2, Figure 3 and Figure 4).

TRPV1 channels are activated by oxidative stress and can disrupt intracellular calcium homeostasis, leading to mitochondrial dysfunction and apoptosis. Our data showing that sevoflurane-induced calcium elevation was significantly suppressed by capsazepine supports this mechanism. The increase in mitochondrial depolarization and caspase-9 activity in sevoflurane-treated cells further indicates that the intrinsic apoptotic pathway is engaged, likely through calcium-mediated mitochondrial permeability transition. These findings align with the established role of TRP channels in integrating oxidative stress signals with cell death pathways. Our results demonstrate that ascorbic acid pretreatment significantly attenuated sevoflurane-induced apoptosis, ROS production, and caspase activation in human cardiomyocytes (Figure 3, Figure 4 and Figure 5). This protective effect is consistent with multiple previous studies demonstrating the cardioprotective properties of vitamin C, which mitigates against doxorubicin-induced cardiotoxicity by maintaining mitochondrial integrity and reducing oxidative damage at the subcellular level [12]. Similarly, Kim et al. showed that vitamin C reduces intracellular ROS levels and maintains cellular homeostasis in human pluripotent stem cell-derived cardiomyocytes [13]. More recently, Xu et al. (2025) comprehensively reviewed the molecular mechanisms by which vitamin C exerts cardiovascular protection, emphasizing its role in reducing oxidative stress and restoring endothelial function [14].

Our study adds to this body of evidence by demonstrating that ascorbic acid not only reduces ROS but also modulates TRPV1 channel activity. The observation that ascorbic acid alone reduced basal calcium levels and ROS production compared to control, and that capsazepine further reduced ROS in ascorbic acid-treated cells, suggests that vitamin C may directly or indirectly inhibit TRPV1 channel activity (Figure 2 and Figure 4). The finding is novel, as the interaction between ascorbic acid and TRPV1 channels in cardiomyocytes has not been previously described. Another key finding of our study is the partial reversal of ascorbic acid’s mitigating effects by capsazepine in cells exposed to sevoflurane. In the sevoflurane + ascorbic acid + capsazepine group, we observed further reductions in calcium levels, ROS levels, apoptosis, and caspase activity compared with the sevoflurane + ascorbic acid group (Figure 2, Figure 3, Figure 4 and Figure 5). This suggests that while ascorbic acid effectively suppresses sevoflurane-induced TRPV1 activation, additional TRPV1 blockade provides incremental protection. This observation indicates that the uplifting effect of ascorbic acid is partially mediated through TRPV1 modulation, but that other antioxidant and non-antioxidant mechanisms may also contribute.

The relationship between ascorbic acid and TRPV1 channels has been explored in other contexts. Ascorbic acid induces necrosis in laryngeal squamous cell carcinoma cells through ROS, PKC, and calcium signaling, indicating that the effects of vitamin C on TRP channels may be cell-type specific and concentration-dependent [15]. In our study, ascorbic acid at a concentration of 1 mM was mitigating, whereas higher concentrations might have different effects. Additionally, the epigenetic effects of vitamin C described by Brabson et al. and Kim et al. suggest that longer-term effects on TRPV1 expression could also be relevant, potentially contributing to sustained modulation of oxidative stress responses and apoptotic pathways in cells [16,17].

The clinical relevance of our findings is supported by studies examining vitamin C supplementation in cardiac surgery patients. Hill et al. reviewed the evidence for vitamin C to improve organ dysfunction in cardiac surgery patients and noted that antioxidant supplementation may reduce postoperative complications [18]. Similarly, early administration of vitamin C, hydrocortisone, and thiamine was associated with improved outcomes in patients with septic cardiomyopathy [19]. Our mechanistic findings provide a potential molecular basis for these clinical observations, suggesting that TRPV1 channel modulation may contribute to the cardioprotective effects of vitamin C in the perioperative period (Figure 3).

The role of oxidative stress in sevoflurane-induced myocardial injury has also been investigated in animal models. Akolkar et al. demonstrated that vitamin C mitigates oxidative/nitrosative stress and inflammation in doxorubicin-induced cardiomyopathy, supporting the broader concept that vitamin C can protect against diverse forms of oxidative cardiac injury [20]. Vitamin C exhibited activity against LPS-induced septic cardiomyopathy through downregulation of oxidative stress and inflammation [21]. Our study complements these findings by identifying a specific ion channel mechanism through which oxidative stress leads to cell death and by which vitamin C mitigates/damage-reduces this effect.

While our results consistently demonstrate the protective effects of ascorbic acid, some studies have reported pro-oxidant effects of vitamin C under certain conditions. Baek et al. showed that ascorbic acid can induce necrosis in cancer cells by generating ROS, highlighting the context-dependent nature of vitamin C’s effects [15]. In our study, the 1 mM concentration and 30 min pretreatment protocol were clearly protective, but different concentrations or exposure durations might yield different results. Additionally, our in vitro model using cultured human cardiomyocytes may not fully replicate the complex in vivo environment, including the presence of other cell types, neurohumoral factors, and the pharmacokinetics of sevoflurane and ascorbic acid. Another consideration is that we used capsazepine as a TRPV1 antagonist, but its specificity has been questioned in some studies. While capsazepine is widely accepted as a selective TRPV1 antagonist, off-target effects cannot be completely excluded. Future studies using genetic approaches, such as TRPV1 knockdown or knockout, would provide more definitive evidence for the role of these channels.

### Clinical Implications

Our findings have several potential clinical implications. First, they suggest that monitoring and managing oxidative stress in patients undergoing sevoflurane anesthesia may be important for myocardial protection, particularly in high-risk cardiac-surgery patients. Second, the demonstration that ascorbic acid mitigates against sevoflurane-induced cardiotoxicity supports the potential role of vitamin C supplementation in the perioperative period. The review by Hill et al. provides a pragmatic approach to vitamin C administration in cardiac surgery patients, and our mechanistic findings support such clinical strategies [18]. Third, the involvement of TRPV1 channels suggests that these channels may represent a therapeutic target for preventing anesthetic-induced myocardial injury, although the potential hemodynamic effects of TRPV1 modulation would need careful evaluation.

Limitations: While apoptosis was primarily assessed using APOPercentage dye uptake, caspase-3 enzyme activity was also measured as a supportive indicator; however, the absence of additional confirmatory assays (e.g., Annexin V/PI or TUNEL) remains a limitation of the study.

## 5. Conclusions

In conclusion, this in vitro study demonstrates that exposure to sevoflurane is associated with increased apoptosis in human cardiomyocytes under the conditions tested. This effect appears to involve ROS-mediated activation of TRPV1 channels, leading to calcium overload, mitochondrial dysfunction, and caspase activation. Ascorbic acid pretreatment reduced these markers of cellular injury, an effect partially reversed by TRPV1 antagonism, suggesting that vitamin C may protect, at least in part, by modulating TRPV1 channel activity. These findings provide new insights into potential molecular mechanisms underlying sevoflurane-associated cardiotoxicity and identify TRPV1 channels as a candidate therapeutic target warranting further investigation. However, we emphasize that these are proof-of-concept findings from an isolated cell system; clinical translation requires in vivo validation and human trials. Future studies should explore dose–response relationships of ascorbic acid, longer-term effects of TRPV1 modulation on myocardial function, and the relevance of these mechanisms at clinically relevant sevoflurane concentrations.

## Data Availability

The original contributions presented in this study are included in the article. Further inquiries can be directed to the corresponding author.

## Abbreviations

Ca^2+^	Calcium ions
Cap	Capsaicin
Cpz	Capsazepin
Sevo	Sevoflurane
AA	Ascorbic acid
Mitoc. Dep	Mitochondrial depolarization
ROS	Reactive oxygen species
HC	Human cardiomyocyte
HCCs	Human cardiomyocyte cells
TRP	Transient receptor potential
TRPV1	Transient receptor potential vanilloid 1
